# Bionomics of Anopheline species and malaria transmission dynamics along an altitudinal transect in Western Cameroon

**DOI:** 10.1186/1471-2334-10-119

**Published:** 2010-05-19

**Authors:** Timoléon Tchuinkam, Frédéric Simard, Espérance Lélé-Defo, Billy Téné-Fossog, Aimé Tateng-Ngouateu, Christophe Antonio-Nkondjio, Mbida Mpoame, Jean-Claude Toto, Thomas Njiné, Didier Fontenille, Herman-Parfait Awono-Ambéné

**Affiliations:** 1 Laboratory of Applied Biology and Ecology (LABEA), Department of Animal Biology, Faculty of Sciences of the University of Dschang, PO Box 067 Dschang, Cameroon; 2 Laboratoire de Recherche sur le Paludisme, Organisation de Coordination pour la lutte contre les Endémies en Afrique Centrale (OCEAC), BP 288 Yaoundé, Cameroon; 3 Hydrobiology laboratory, Faculty of Sciences of the University of Yaounde I, PO Box 812 Yaounde, Cameroon; 4 Laboratoire de Lutte contre les Insectes Nuisibles (LIN-UR 016), Institut de Recherche pour le Développement (IRD), 911 Av Agropolis, BP 64501, 34394 Montpellier, France

## Abstract

**Background:**

Highland areas of Africa are mostly malaria hypoendemic, due to climate which is not appropriate for anophelines development and their reproductive fitness. In view of designing a malaria control strategy in Western Cameroon highlands, baseline data on anopheline species bionomics were collected.

**Methods:**

Longitudinal entomological surveys were conducted in three localities at different altitudinal levels. Mosquitoes were captured when landing on human volunteers and by pyrethrum spray catches. Sampled *Anopheles *were tested for the presence of *Plasmodium *circumsporozoite proteins and their blood meal origin with ELISA. Entomological parameters of malaria epidemiology were assessed using Mac Donald's formula.

**Results:**

Anopheline species diversity and density decreased globally from lowland to highland. The most aggressive species along the altitudinal transect was *Anopheles gambiae *s.s. of S molecular form, followed in the lowland and on the plateau by *An. funestus*, but uphill by *An. hancocki*. *An. gambiae *and *An. ziemanni *exhibited similar seasonal biting patterns at the different levels, whereas different features were observed for *An. funestus*. Only indoor resting species could be captured uphill; it is therefore likely that endophilic behaviour is necessary for anophelines to climb above a certain threshold. Of the ten species collected along the transect, only *An. gambiae *and *An. funestus *were responsible for malaria transmission, with entomological inoculation rates (EIR) of 90.5, 62.8 and zero infective bites/human/year in the lowland, on the plateau and uphill respectively. The duration of gonotrophic cycle was consistently one day shorter for *An. gambiae *as compared to *An. funestus *at equal altitude. Altitudinal climate variations had no effect on the survivorship and the subsequent life expectancy of the adult stage of these malaria vectors, but most probably on aquatic stages. On the contrary increasing altitude significantly extended the duration of gonotrophic cycle and reduced: the EIR, their preference to human blood and consequently the malaria stability index.

**Conclusion:**

Malaria epidemiological rooting in the outskirts of Western Cameroon highlands evolves with increasing altitude, gradually from stable to unstable settings. This suggests a potential risk of malaria epidemic in highlands, and the need for a continuous epidemiological surveillance.

## Background

Highland areas of Africa are known to be malaria hypoendemic, due to climate (low temperatures and relative humidity), which is not appropriate for anophelines development and their reproductive fitness [1]. Nevertheless, the probability that an entomological inoculation is effective (the success probability) in these regions of low transmission is higher than in holoendemic areas [2]. In fact, the highest disease risks are observed among populations exposed to low-to-moderate intensities of transmission, and mean age of disease patients increases with decreasing transmission intensity [3].

For the past decades, there has been an increase in the number of malaria epidemics, and the question is raised of the boosting of malaria transmission in African high altitude areas as a consequence of global warming [4,5]. Moreover, there is a spread of endemic malaria into the highland fringes [6]. In 2003, it was estimated that 110 million peoples were at risk of malaria epidemics in Africa and 110 000 of these died of the disease [7]. One year later, another survey indicated a higher rate of 155 000-310 000 deaths (out of 12 million malaria episodes) attributable to epidemics [8]. Unlike in lowland environments where the potential for malaria epidemics owing to decreasing levels of natural immunity may be offset by negative impacts of urbanization on anopheline mosquito larvae, and where therefore malaria control may be simpler [9], control strategies in highland areas should be much more based on prevention of epidemics. Tools to predict and forecast malaria epidemics are therefore urgently needed.

Since malaria epidemics appear suddenly and terminate within a few months, detection based solely on increasing incidence and conducted by network of health workers in sentinel stations as applied so far [10,11], although likely to be cost-effective [12], would not provide sufficient time for an adequate response. In fact, epidemics usually require emergency measures that must be implemented as promptly as possible in order to be effective. Moreover, a declaration of an emergency, generally based on the inability of the medical services to cope with demands of patients following malaria outbreaks must be made. Forecasts of epidemics based on indicators preceding elevated infection and disease rates in human population are therefore required.

Recently, modern tools to help decision makers to predict malaria epidemics were proposed. They rely on the use of: combinations of satellite derived climate-based data [13,14], weather monitoring combined with disease surveillance [15], the normalized difference vegetation index (NDVI) [16], the El Niño Southern Oscillation phenomenon (ENSO) [17,18], and soil moisture [19]. Guidelines for implementing some of these models were provided and approved [20,21]; they defined the steps to be taken while setting up a national malaria early warning system (MEWS). Unfortunately, very few African countries have adequately qualified human resources and appropriate meteorological facilities to be able to implement such measures. Consequently, the challenge of developing new tools for malaria prevention in highlands remains.

It was demonstrated that abundance and increased density of indoor resting *Anopheles *vectors were positively correlated with incidence of malaria in the human population one month later, indicating that entomological indicators could be used to predict or confirm incipient epidemics as well [18]. Thus, monitoring parameters of anopheline bionomics and vectorial capacity [ie: abundance (indoor density and biting rate), feeding behaviour, gonotrophic cycle and survivorship], may provide good conclusive early warning of malaria outbreaks.

The hypothesis of our study was that there is a relationship between: altitudinal climate variations and vectorial capacity of anopheline populations. Such variations in malaria transmission parameters lead to different risk levels for malaria infections and epidemics, taking into account the little or no functional immunity in human populations of highlands.

Baseline data and detailed knowledge on the biology of each species of the vectorial system are therefore a prerequisite for implementation of any MEWS. Herein, data on anopheline species diversity and abundance, implication in malaria transmission, their survivorship, host-seeking behavior and subsequent malaria stability index are provided for three sites in Western Cameroon, spread out over an altitudinal transect, starting from a lowland plain, across a forest sheer cliff, then a highland plateau till a hilly landscape.

## Methods

### Study areas

**Figure 1:**
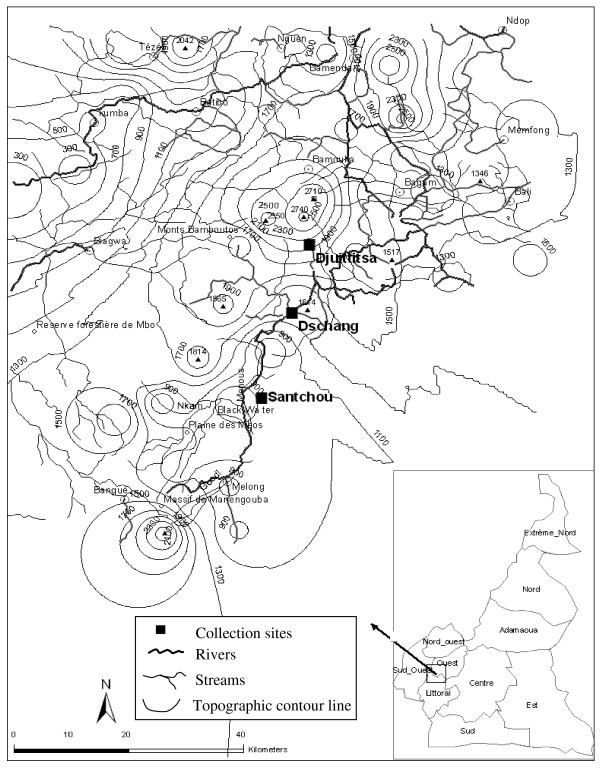
**Topographic map of the Menoua Division in Cameroon showing the geographical localizations of the collection sites**.

The study was carried out in the Menoua Division, an outskirts of the Western-Cameroon Highlands. It is a peculiar zone as far as topography and climate are concerned, located in a savannah landscape within the Guineo-Congolese bioclimatic domain, on the Cameroon Volcanic Line. Four seasons can be distinguished as follows: the main dry season (MDS: November to mid-March), the small rainy season (SRS: mid-March to May), the small dry season (SDS: June to July) and the main rainy season (MRS: August to October) [22]. Three collection sites were selected along an altitudinal transect ranging from 750 to 1965 m above the sea level (Figure 1).

The village of Santchou (5°15'N; 9°50'E) is located at an altitude of 750 m within the wide Mbô plain, whose landscape is a shrubby savannah with some isolated woodland along the streams, limited towards Northeast by a sloppy forest cliff about 14 km from Santchou. The river Nkam and its tributaries provide a dense hydrography and the swampy plain floods frequently during the rainy seasons. The annual average temperature is 28 ± 3°C with daily thermal amplitude of less than 10°C. Rains are abundant with annual average rainfalls of 2200 mm.

On top of this sheer cliff, at an altitude of 1400 m on the Bamiléké plateau, lies the city of Dschang (5°27'N; 10°04'E), located at 22 km from Santchou. The topography is characterised by the juxtaposition of small hills furrowed by little streams flowing down towards swampy lakes around which indoor mosquito collections were conducted. The average annual temperature is 20.5 ± 6°C, with February being the hottest month. The daily thermal amplitude can exceed 13°C during the dry season, and constitutes a peculiarity of this locality. The mean annual rainfall is 2000 mm.

Finally, Djuttitsa (5°36'N; 10°05'E) is located approximately 20 km away from Dschang towards the mountains at an altitude of 1965 m. The village is surrounded by: industrial tea plantations belonging to the Cameroon Development Corporation (CDC) and the Djuttitsa Tea Estate (DTE), the experimental land of the National Research Institute of Agronomy for Development (IRAD) for production of potato seeds and a wide pasture for cattle. The average annual temperature is 16.5 ± 7°C and the mean annual rainfall is 1600 mm.

### Mosquito collections and field processing of anophelines

Longitudinal monthly entomological surveys were conducted in each study site. Adult mosquitoes were captured by Human Landing Catches (HLC) and Pyrethrum Spray Collections (PSC).

Night HLC were conducted in 6 houses per site (selected from the mosquito yield of preliminary surveys), by teams of volunteers who had received training for mosquito collections. The first team was installed from 06.00 pm to 01.00 am and the second from 01.00 am to 06.00 am, to avoid sampling fatigue. Specimens collected were maintained in a cooler containing ice blocks. They were identified individually under binocular, using morphological identification keys [23,24]. A subset was dissected for examination of ovarian tracheoles and determination of parity as previously described [25].

Day-time PSC were conducted by spraying pyrethrum insecticide in houses, outdoor resting shelters and cowsheds of compounds which were selected based on their mosquito yield and the acceptability of inhabitants to cooperate during a preliminary survey. Dead or fainted mosquitoes were collected in test tubes. Culicinae were discarded while Anophelinae were sorted and also identified as mentioned above for night collections. Female mosquitoes were classified according to their repletion status into unfed, freshly fed, half-gravid and gravid specimens. Blood fed mosquitoes had their blood meal squashed on Whatman N°1 filter paper and stored dry with a desiccant at -20°C until testing for blood meal origin. The rest of mosquito carcass was stored in individually labeled test tubes at -20°C.

The fed/gravid ratio was determined for each species at the different sites and used to estimate the duration of gonotrophic cycle. For this calculation, half-gravid and gravid mosquitoes were grouped. Determination of the duration of gonotrophic cycle was based on the assumption that mortality rate is constant throughout the ovarian cycle. In this case, the fed/gravid ratio is approximately 1:1 for a gonotrophic cycle of 2-days, whereas it would be 1:2 for a 3-days cycle, because of an additional inserted day for half-gravid status [26]. The daily survival rate (*p*) of anopheles was then determined for each altitudinal site of the transect, using Davidson's formula [27]:

(where *x *is the duration of the gonotrophic cycle in days, *Np *and *Nn *the numbers of parous and nulliparous females respectively in the population). Life expectancy estimates (*EL*) were thereafter calculated using Mac Donald's formula [28]:

### Laboratory analysis of mosquitoes

The head and thorax of female anopheles were separated from the rest of the body and tested for the presence of circumsporozoite protein (CSP) of *Plasmodium falciparum *Welch with ELISA [29,30]. *Plasmodium malariae *Laveran and *Plasmodium ovale *Stephens were not tested since they are present in low percentage in Cameroon compared to *P. falciparum*, while *Plasmodium vivax *Grassi & Feletti was completely absent. The CSP rate and the 95% CI were calculated. The entomological inoculation rate (EIR) was thereafter determined by multiplying the annual human biting rate (HBR) by the mean CSP rate for each species at the different sites.

Blood components from the spots on filter papers were eluted in normal saline buffer overnight and the origins of blood meals determined with an ELISA test [31]. The technique identified the source of blood meals as from human, bovine, ovine (sheep or goat), equine (horse or donkey), pig or avian (chicken) host. The feeding preference of anophelines was assessed by calculating the human blood rate (HR) as the ratio of blood from human origin to the total number of blood spots analyzed. This enabled the calculation of the anthropophilic index (*a*) as the ratio of the HR to the duration of the gonotrophic cycle in days. The stability index (*St*) of malaria at the different altitudinal sites was thereafter determined using Mac Donald's formula [28].

The DNA of *Anopheles gambiae *s.l. specimens were extracted from their legs that have been conserved dry in freezer at -20°C, then amplified in enzymatic polymerised chain reaction (PCR) of a gene (from the IGS region) which codes for ribosomal RNA (rRNA) and whose length varies within the species members of the complex [32]. This PCR-rDNA enabled identification of the sibling species as either *An. gambiae sensus stricto*, *An. arabiensis *or *An. melas*, by comparison with a marker of molecular weight. Another PCR, a restricted fragment length polymorphism (PCR-RFLP) was performed on all the specimens diagnosed as *An. gambiae *s.s., to distinguish between molecular forms M and S [33,34].

### Ethical issues

A national ethical clearance (N°: FWA IRB00001954, dated 11/05/2005) was obtained from the National Ethics Committee (Yaounde, Cameroon) and an institutional ethical approval from OCEAC (N°: 0287-05/SG/CAB, dated 17/03/2005). The various aspects of the work were conducted in collaboration with the local Health District Authorities. The free informed consents of volunteers (to participate in mosquito collections) and head of families were requested through individual discussions and group meetings, prior to the enrolment of their house in the study. For those willing to cooperate, presumptive malaria treatment was given throughout the course of the study as recommended by the National Malaria Control Programme.

## Results

### Anopheline species diversity and abundance: implications for malaria transmission

A total of 20 surveys were carried out from April 2003 to March 2005 in each site. Table 1 shows the number of specimens collected by HLC throughout the study and gives the human biting rate (HBR) of each species along the altitudinal transect. A total of 1787 (14.9 bites per human per night), 1079 (9.0 b/h/n) and 40 (0.3 b/h/n) anopheles specimens were collected in the lowland plain, the highland plateau and the hill site respectively. In the lowland at Santchou, eight anthropophagous *Anopheles *species were identified on morphological grounds; these included *Anopheles gambiae *(11.5 b/h/n) and *An. funestus *(0.9 b/h/n). In addition to these species, *An. hancocki *and *An. moucheti *were also found in Dschang. The biting densities at this altitude were 7.1 and 1.1 b/h/n for *An. gambiae *and *An. funestus *respectively. At Djuttitsa, only two anopheline species were recorded and their densities were very low: *An. gambiae *(0.1 b/h/n) and *An. hancocki *(0.2 b/h/n). The most frequent anopheline species along the whole transect was *An. gambiae *(77.4%), followed by *An. funestus *(7.9%). There was homogeneity in the composition of *An. gambiae *complex, as all specimens along the transect were detected to be *An. gambiae *sensus stricto of the S molecular form.

**Table 1:** Biodiversity and abundance of human-landing anopheles captured in the three study sites along the altitudinal transect

	Study sites of the altitudinal transect ^a^						
	
Anopheles species	Santchou (750 m)		Dschang (1400 m)		Djuttitsa (1965)		Overall study sites
				
	HBR (NoCSP)	PR (NoDEO)	HBR (NoCSP)	PR (NoDEO)	HBR (NoCSP)	PR (NoDEO)	NoCOL (%)
*An coustani*	0.1 (9)	-	0 (3)	-	0 (0)	-	12 (0.4)
*An funestus*	0.9 (104)	69.2 ± 17.7 (26)	1.1 (126)	63.7 ± 7.2 (102)	0 (0)	- (0)	230 (7.9)
*An gambiae*	11.5 (1380)	74.5 ± 3.3 (687)	7.1 (857)	66.8 ± 3.1 (722)	0.1 (12)	- (0)	2249 (77.4)
*An hancocki*	0 (0)	-	0.1 (14)	-	0.2 (28)	- (0)	42 (1.4)
*An moucheti*	0 (0)	-	0.1 (12)	-	0 (0)	-	12 (0.4)
*An nili*	0.4 (45)	-	0.1 (14)	-	0 (0)	-	59 (2.0)
*An paludis*	0.5 (62)	-	0.2 (18)	-	0 (0)	-	80 (2.8)
*An pharoensis*	0.1 (7)	-	0 (3)	-	0 (0)	-	10 (0.3)
*An wellcomei*	0.1 (14)	-	0.1 (6)	-	0 (0)	-	20 (0.6)
*An ziemanni*	1.4 (166)	-	0.2 (26)	-	0 (0)	-	192 (6.6)
**Total anopheline mosquitoes**	**14.9 (1787)**	**74.3 ± 3.2 (713)**	**9.0 (1079)**	**66.4 ± 2.9 (824)**	**0.3 (40)**	**- (0)**	**2906**

^**a **^Mosquitoes were collected at each of the three study sites by a total of 120 human-nights.

HBR: Human-biting rates expressed in bites per man per night (b/h/n); NoCSP: Number of female *Anopheles *mosquitoes examined for the presence of circum-sporozoite protein (CSP); PR: Parous rate ± 95% confidence interval; NoDEO: Number of female *Anopheles *specimens whose ovaries were dissected and examined; NoCOL: Number of female *Anopheles *mosquitoes collected.

**Figure 2:**
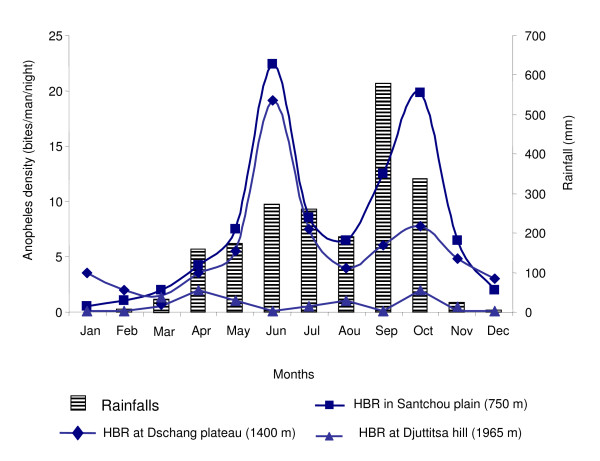
**Spatial-temporal variations of human biting rate (HBR) for the overall anopheline species**. The means of monthly rainfalls and biting densities for the two years of survey were determined and considered, as the study area maintained similar climatic conditions during these two consecutive years

**Figure 3:**
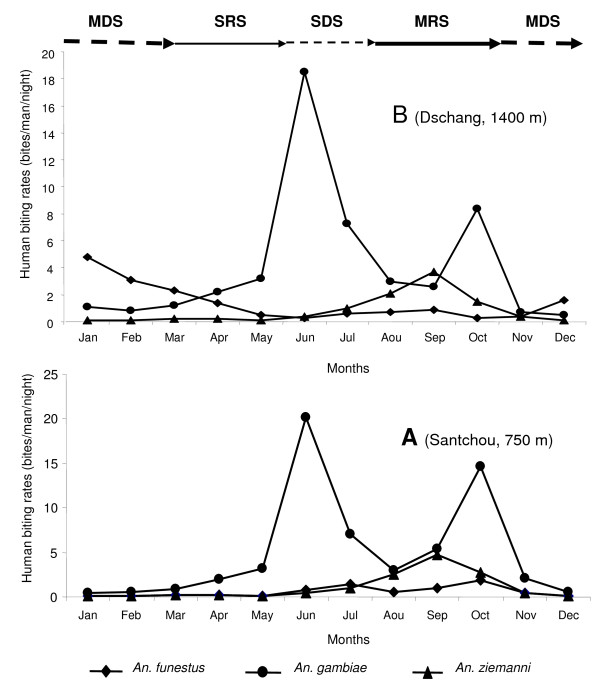
**Seasonal variations of the most frequent anopheline species' biting densities in the lowland plain of Santchou (A) and the plateau of Dschang (B)**. The periods of high malaria risk are: the months of June and October in the plain, and the months of January, June and October on the plateau site. MDS: main dry season, SRS: small rainy season, SDS: small dry season, MRS: main rainy season.

Figure 2 shows the spatial-temporal variations of the overall anopheline species' biting densities. The population dynamic of the overall anopheline species in the lowland was closely associated with rainfalls and presented two peaks of aggressiveness: at the beginning of SDS (June) and the end of MRS (October). Similar profile was observed on the plateau but with a lower HBR in October. Figure 3 displays the seasonal variations of anopheline species density in Santchou (A) and Dschang (B). It indicates the similar biting patterns of *An. gambiae *and *An. ziemanni *in both sites, with two peaks (June and October) for *An. gambiae *and a single peak (September) for *An. ziemanni*. However, different seasonal variation features were observed in the two sites for *An. funestus*; with a biting peak occurring only on the plateau site and during the MDS.

Of the ten anopheles species recorded along the transect, only *An. gambiae *and *An. funestus *of the plain and the plateau sites bore *Plasmodium *CSP. No mosquito was found uphill with *Plasmodium *CSP. Table 2 gives the spatial variations of the EIR for these two malaria vectors. In Santchou and Dschang, the main malaria vector was *An. gambiae*, followed by *An. funestus *for which a higher CSP rate was reported on the plateau compared to the lowland. The level of overall malaria transmission decreased from 90.5 infective bites per human per year (ib/h/yr) in the holoendemic lowland plain, to 62.8 ib/h/yr on the mesoendemic highland plateau and finally to zero ib/h/yr uphill where malaria is hypoendemic.

**Table 2:** Malaria transmission levels and involvement of anopheline species in the three study sites along the altitudinal transect

	Study sites of the altitudinal transect ^a^					
	
	Santchou (750 m)		Dschang (1400 m)		Djuttitsa (1965 m)	
			
Anopheles species	CSP rate ± 95% CI (NoT)	EIR (ma)	CSP rate ± 95% CI (NoT)	EIR (ma)	CSP rate ± 95% CI (NoT)	EIR (ma)
*An funestus*	1.0 ± 2.0 (96)	3.3 (328.5)	4.2 ± 3.6 (119)	16.1 (383.3)	- (0)	- (0)
*An gambiae*	2.1 ± 0.8 (1362)	88.1 (4197.5)	1.8 ± 1.0 (840)	46.9 (2606.7)	0 (10)	0 (36.5)
**Total malaria vectors**	**2.0 ± 0.7 (1458)**	**90.5 (4526)**	**2.1 ± 1.0 (959)**	**62.8 (2990.0)**	**0 (10)**	**0 (36.5)**

^**a **^Mosquitoes were collected from each of the three study sites by a total of 120 human-nights.

NoT: Number of female *Anopheles *mosquitoes tested with ELISA Circum-sporozoite protein (CSP); ma: Biting rates estimated by number of bites/human/year (b/h/yr); EIR: Entomological inoculation rates estimated as number of infected bites per human per year (ib/h/yr).

Table 3 shows the results of PSC which were conducted in more than 200 houses and shelters per study site. Anopheles specimens were seldom captured in outdoor shelters in all the sites. Only *An. gambiae, An. funestus *and *An. hancocki *were sampled indoor. *An. gambiae *and *An. funestus *were collected in the lowland area with an average density of 3.1 per house (/he), whereas all the three species were found on the plateau with an overall mean density of 0.9 /he. Some scarce specimens of *An. gambiae *and *An. hancocki *could be found at the hill site at about 0.2 /he. Statistical analysis of indoor anopheles densities by H test of Kruskal-Wallis showed that there was a highly significant reduction of the anopheles density with increasing altitude (H = 209.46, p < 10^-4^). The difference in anopheles densities with seasons was also very significant (H = 87.68, p < 10^-4^), and for all study sites the maximum density was recorded in the SDS or end of MRS while the minimum density occurred during the MDS, just as for the HLC.

**Table 3:** Abundance, repletion status and gonotrophic cycles of indoor-resting anopheles along the altitudinal transect

Study sites of the altitudinal transect	Number of houses sprayed ^a^	Anopheline species collected	ADH ^b ^(No collected) ^c^	No unfed / No fully blood-fed / No half or fully gravid ^c^	Fed/gravid ratio	Estimated duration of gonotrophic cycle (days)
Djuttitsa (1965 m)	210	*An funestus*	0 (0)	- / - / -	-	-
		*An gambiae*	0.18 ± 0.39 (38)	20 / 3 / 16	1 : 5.33	6 to 7
		*An hancocki*	0.06 ± 0.23 (12)	3 / 2 / 7	1 : 3.5	4 to 5
		**Total**	**0.24 ± 0.47 (50)**	**23 / 5 / 23**	**1 : 4.42**	**4 to 7**
	
Dschang (1400 m)	224	*An funestus*	0.07 ± 0,29 (16)	3 / 3 / 10	1 : 3.33	4 to 5
		*An gambiae*	0.83 ± 1.21 (186)	90 / 25 / 71	1 : 2.84	3 to 4
		*An hancocki*	0.02 ± 0.13 (4)	1 / 1 / 2	1 : 2.0	3
		**Total**	**0.92 ± 1.26 (206)**	**94 / 29 / 83**	**1 : 2.72**	**3 to 5**
	
Santchou (750 m)	259	*An funestus*	0.19 ± 0.51 (48)	35 / 4 / 9	1 : 2.25	3 to 4
		*An gambiae*	2.88 ± 2.78 (746)	455 / 98 / 193	1 : 1.97	2 to 3
		*An hancocki*	0 (0)	- / -. / -	-	-
		**Total**	**3.06 ± 2.89 (794)**	**490 / 102 / 202**	**1 : 2.11**	**2 to 4**

^a ^About 10 houses were sprayed in each of the three study sites of the altitudinal transect during every survey, assigning our protocol to the Minimum Sample Size Approach (MSSA) [42]. However, houses were not randomly sampled, but biased by the tendency of targeting and spraying repeatedly those houses where previous enquiries were positive. Results obtained for average mosquito densities were therefore overestimated.

^b ^ADH: Average female *Anopheles *density /house ± standard deviation.

^c ^No: Number of female *Anopheles *mosquitoes.

### Host feeding preference and duration of the gonotrophic cycle

A total of 284 blood meal spots from Santchou, 85 from Dschang and 19 from Djuttitsa were examined with ELISA for host determination (Table 4). The proportion of blood meals from human host (HR) was 96.8% in Santchou, 85.9% in Dschang and 57.9% in Djuttitsa (χ^2 ^= 45.99; d.f. = 2; p < 10^-8^); indicating a significant reduction in the human blood rate (HR) from the lowland to the highland. All the three anthropophagous anopheline species sampled by PSC (e.g., *An. gambiae*, *An. funestus *and *An. hancocki*) could occasionally feed on alternative available vertebrate hosts.

**Table 4:** Blood feeding preferences of *Anopheles *species at the different study sites of the altitudinal transect

	Blood meal sources at the study sites of the altitudinal transect ^a^			
		
Vertebrate hosts	Santchou (750 m)	Dschang (1400 m)	Djuttitsa (1965 m)	Overall study sites (%)
Human	275 (5 f + 270 g)	73 (4 f + 69 g)	11 (9 g + 2 h)	359 (92.5%) (9 f + 348 g + 2 h)
Chicken	5 (1 f + 4 g)	5 (2 f + 3 g)	2 (g)	12 (3.1%) (3 f + 9 g)
Pig	3 (3 g)	3 (1 f + 2 g)	0	6 (1.5%) (1 f + 5 g)
Horse	0	2 (1 g + 1 h)	3 (2 g + 1 h)	5 (1.3%) (3 g + 2 h)
Ovine	0	2 (2 g)	1 (g)	3 (0.8%) (3 g)
Bovine	0	0	2 (g)	2 (0.5%) (2 g)
Dog	0	0	0	0
Mixte (Chicken+Human)	1 (g)	0	0	1 (0.3%) (g)

Total number of blood meals tested	284 (6 f + 278 g)	85 (7 f + 77 g + 1 h)	19 (16 g + 3 h)	388 (13 f + 371 g + 4 h)
**Human blood rate (HR)**^b ^**× 100**	**96.8%**	**85.9%**	**57.9%**	**92.5%**

^a ^The numbers in brackets indicate the distribution of blood meals among the different anopheline species (f: blood meals of *An. funestus*; g: blood meals of *An. gambiae*; h : blood meals of *An. hancocki*)

^b ^The human blood rate (HR) expressed as the ratio of blood meals from humans to the total blood samples examined with ELISA.

The fed/gravid ratio for *An. funestus *was 1:2.25 and 1:3.33 in the lowland plain and the highland plateau respectively, suggesting that the duration of gonotrophic cycle was 3-4 days in Santchou and 4-5 days Dschang. For *An. gambiae*, the ratio was 1:1.97, 1:2.84 and 1:5.33 in Santchou, Dschang and Djuttitsa respectively; indicating that the duration of gonotrophic cycle for this species was 2-3 days in the lowlands, 3-4 days on the plateau and 6-7 days uphill. At equal altitude, the duration of gonotrophic cycle was always one day shorter for *An. gambiae *compared to *An. funestus *(Table 3).

### Life expectancy of anopheles and malaria stability

Dissections of ovarian tracheoles indicated an overall parous rate of 74.3% in Santchou and 66.4% in Dschang (Table 1). There was a reduction in the parous rate of the malaria vector populations between Santchou and Dschang, which was statistically significant for *An. gambiae *(χ^2 ^= 10.22, p < 0.001). Despite the increase in duration of the gonotrophic cycle with altitude, the average daily survival rates (*p*) of *An. funestus *in the lowland and the highland plateau were similar and equal to 0.90 due to a higher parity rate in the plain. Likewise, the mean daily survivorships of *An. gambiae *at Santchou and Dschang were the same and equal to 0.89. As a matter of fact, the expected durations of life for adult stage (*EL*) were similar at the different altitudinal sites: 9.49 days for *An. funestus *and 8.58 days for *An. gambiae *(Table 5). The sample size of specimens from Djuttitsa was too low to allow this analysis. Malaria stability index (*St*) was 3.43 in Santchou and 2.04 in Dschang, indicating stable and intermediate situations for the disease in the respective localities according to an established scale [28].

**Table 5:** Relationship between altitude, life expectation of the potential malaria vectors and malaria stability

Study sites of the altitudinal transect	Anopheline species	Median length of the gonotrophic cycle (x) (Range)	Daily survival rates (p) / Life expectancies (EL)	Human blood rate (HR) / Anthropo-philic index (a)	Malaria stability index (St) ^a^
Djuttitsa (1965 m)	*An funestus*	-	- / -	- / -	-
	*An gambiae*	6.5 (6-7)	- / -	47.4% / -	-
	*An hancocki*	4.5 (4 - 5)	- / -	10.5% / -	-
	**Total**	**5.5 (4 - 7)**	**- / -**	**57.9% / -**	-
Dschang (1400 m)	*An funestus*	4.5 (4 - 5)	0.90 / 9.49	4.7% / 0.010	0.09
	*An gambiae*	3.5 (3 - 4)	0.89 / 8.58	81.2% / 0.232	1.99
	*An hancocki*	3 (2.5 - 3.5)	- / -	- / -	-
	**Total**	**4 (3 - 5)**	**0.90 / 9.49**	**85.9% / 0.215**	**2.04**
Santchou (750 m)	*An funestus*	3.5 (3 - 4)	0.90 / 9.49	1.7% / 0.005	0.05
	*An gambiae*	2.5 (2 - 3)	0.89 / 8.58	95.1% / 0.380	3.28
	*An hancocki*	-	- / -	- / -	-
	**Total**	**3 (2 - 4)**	**0.91 / 10.60**	**96.8% / 0.323**	**3.43**

^**a **^The epidemiology of malaria in the lowland and on the plateau was characterized based on the malaria stability index [28], which determines the rooting of the disease in a particular zone: i)- 0 < St < 0.5 for unstable malaria zones, ii)- 0.5 < St < 2.5 for intermediate zones of malaria stability, and iii)- St > 2.5 for stable malaria zones.

## Discussion and conclusion

HLC were more efficient than PSC in reflecting the biodiversity and density of the anopheline fauna in all study sites, although the latter was carried out in twice as much houses. Several of these species (*Anopheles coustani, An. paludis, An. pharoensis, An. wellcomei*, and *An. ziemanni*) were frequently caught indoor by HLC, but never sampled by PSC. It is likely that they were outdoor resting species. Investigations should be conducted to unveil their resting behaviour which should be included in the strategic planning of malaria control in highlands. In fact, they might be secondary malaria vectors, for they were found carrying *Plasmodium *antigens in the forest zone of South-Cameroon, although at very low rates [35-39]. However, they were scarce even outdoor (data not shown), compared to what is usually found in the above other cited areas of the country where microclimatic conditions are favourable, suggesting that the hostile climate in highlands restricts outdoor resting opportunities to very few hidden sites.

There was a qualitative drop in anopheline species diversity with increasing altitude, although two additional species were found on the plateau. The availability of permanent breeding sites in the valleys of Dschang such as lakes and swamps, suitable for the development of *An. funestus *but also *An. moucheti *and *An. hancocki *larvae, could justify the presence of these two latter species rather than altitude. It is likely that up to a certain level, availability of convenient larval habitats attenuates the suppressive effect of altitude and/or climate on the mosquito biodiversity. This antagonistic effects might have limited the number of sibling species of *An. gambiae *complex along the transect to a single one. Moreover, only the indoor resting species found in lowland could be captured uphill. These same species were those found at the elevated areas of the Mount Cameroon region (another highland zone in south-west of the country), where evidence for implication of *Anopheles hancocki *in malaria transmission was shown [40,41]. It is likely that endophilic behaviour is necessary for anophelines to climb above a certain threshold, which in the case of Western-Cameroon is situated beyond the Bamiléké plateau at 1400 m. This observation helps to understand why the idea of using indoor resting anopheles to predict highland malaria epidemics was put forward [42].

Likewise, a quantitative drop in the density of anopheles with altitude was recorded both for HLC and PSC, also probably due to altitudinal climate variations, which empirically was incriminated to reduce their survivorship and reproductive fitness. Apart from *An. hancocki*, which seemed to be adapting to highland conditions, vector densities from HLC declined across the sheer cliff (650 m height), by 1.7-fold, then rapidly by 30-fold till uphill (465 m height). However, densities of samples from PSC followed a more regular decreasing slope of about 3.5-fold along the transect. This spatial evolution of the densities indicated a moderate decline rate compared to the 50% per 86 m rise in altitude observed in Mount Kilimanjaro [26].

At equal altitude, the maximum density of mosquitoes was always recorded in the SDS and the minimum in the MDS, probably due to both the availability of puddles and the suitability of temperatures. During the MDS, malaria transmission was maintained in Dschang and it was essentially due to *An. funestus*, whose sporozoite rate reached 4.2%. *An. funestus *larvae were found to develop in permanent ponds and lake sides, especially widespread around Dschang city. Absence of such permanent breeding habitats in Santchou resulted in a more seasonal pattern of malaria transmission with a steep drop in biting density of all anopheline species during the MDS. Even the river Nkam which meanders around and then across the village of Santchou did not yield enough amount of *An. nili *as presumed from observations in South-Cameroon [37], probably due to absence of forest ecosystem. A striking increase in biting rate of *An. ziemanni *was noted at the middle of the MRS both in Santchou and Dschang, indicating a relationship between the rainfall and the density of this species.

As in most localities of sub-Saharan Africa, *An. gambiae *and *An. funestus *were the malaria vectors along the transect. *An. funestus *supplemented malaria transmission and bridged the gap to compensate for lack of malaria transmission which is normally induced by the microclimatic conditions of highlands during the dry season. The vectorial system was not as complex as in the South, where particular effective vectors have adapted to specific niches with population peaks occurring at different times to ensure continuous malaria transmission [35,36,38]. There was a global reduction of about 1.5-fold in malaria transmission level, from the holoendemic lowland to the mesoendemic highland plateau, and this EIR rapidly turned to zero in the hypoendemic zone uphill. This confirms the fact that elevated zones are areas of low or absence of malaria transmission [6,43]. The reduction rate was similar to the one observed in a transect on Mount Cameroon, where the transmission intensity also decreased gradually with increasing altitude, though a third anopheline species was involved in malaria transmission [40,41]. This reduction was moderate compared to the decreasing rate at similar altitudes in Tanzania [44,45]. The low reduction rate at which malaria transmission intensity declines with increasing altitude in Cameroon indicates a potential aptitude of *An. gambiae *and *An. hancocki *to climb uphill and adapt to highland environment. So far, annulment of malaria transmission occurred beyond 1400 m.

The human blood rate was the highest, scoring 92.5% of the overall total blood meal analyzed, followed in each site by the most available domestic animal, especially those spending their night indoor. Mixed blood meals were recorded only in the lowland, and the overall rate of blood detected to be from more than one host species was very low (0.3%), compared to the 18% obtained in Senegal [46]. There was a significant reduction in the HR from the plain across the plateau until uphill, indicating that anophelines exhibited a preference for humans to a higher extent in lowlands than in highlands. However, an attempt to separate the effect of host availability and attractiveness from the effect of altitudinal climate changes is necessary, since it was suggested that the degree of anthropophagy of *An. gambiae *has an innate olfactory basis [47].

In the lowlands of tropical forest, the gonotrophic cycle of *An. gambiae *takes 2 to 3 days. This duration was assessed by mark-release-recapture technique, a method feasible only in localities of high biting rate [48,49]; which is not the case in highlands. An attempt to estimate this entomological factor was carried out by a different method, successfully used before in Tanzania [26]. The duration of gonotrophic cycle was consistently found to be one day shorter for *An. gambiae *as compared to *An. funestus *at equal altitude. Although this period of time increased with altitude in both *An. gambiae *and *An. funestus*, there was no effect on the daily survival probability and consequently on life expectancy of both species. In fact, the significant decrease in the parity rate of both species from the lowlands to the highlands was compensated by an increase in the duration of gonotrophic cycle. As a matter of fact, the calculated survivorship and the life expectancies were almost constant with increasing altitude. Thus, the question of how altitude and/or climate influence mosquito population size remains. We argue that the stage-specific effect of altitude and/or climate on the longevity of anopheles is therefore not on the adults, but more likely on aquatic stages whose breeding habitats directly face the altitudinal climate constraints. Adult malaria vectors may be alleviated from the burden of hostile climate in highland sites by their endophilic behaviour, as inside houses had a microclimate warmer than outside. This explains why only indoor resting species were found uphill.

Spatial variations in malaria transmission level indicated lower level on the plateau as compared to the lowland plain, and complete absence of transmission on the hill site. The malaria immune status of the local human populations therefore decreases with increasing altitude. Although there was no epidemic in these highland sites for the two years of survey, data on the indoor resting density and malaria stability, highlight the fact that this highland area of Western-Cameroon is prone to malaria outbreak in case of climate change. In fact, a density of 0.25 *An. gambiae*/he was said to be associated with epidemic transmission in East-Africa [42], and stability index between 0 and 0.5 to characterize unstable malaria settings [28]. Epidemiological studies based on longitudinal entomological and parasitological surveys, associated to clinical surveillance are ongoing in the same sites in order to establish the threshold values and ranges associated with an eventual epidemic in Western-Cameroon highlands.

## Competing interests

The authors declare that they have no competing interests.

## Authors' contributions

TT conceived and planned the study and its design. He monitored the field and laboratory studies, analyzed and interpreted the data, and drafted the manuscript. DF, FS, MM and TN contributed to the conception of the study and coordinated the laboratory studies. ELD and BTF were Master students and were involved in field and laboratory work for acquisition of data. JCT, CAN and HPAA assisted in mosquito identifications, and other field and laboratory processing. All authors read and approved the final manuscript.

## Pre-publication history

The pre-publication history for this paper can be accessed here:

http://www.biomedcentral.com/1471-2334/10/119/prepub
